# mSphere of Influence: Damage in RNA virus infection—a feature or a bug?

**DOI:** 10.1128/msphere.00255-26

**Published:** 2026-07-10

**Authors:** Katherine C. Barnett

**Affiliations:** 1 Department of Microbiology and Immunology, University of Michigan Medical School 12266, Ann Arbor, Michigan, USA; Virginia-Maryland College of Veterinary Medicine, Blacksburg, Virginia, USA

**Keywords:** RNA virus, innate immunity, pattern recognition receptors, damage-associated molecular patterns, cell death, host-pathogen interactions

## Abstract

Katherine C. Barnett studies cell death and damage in viral infections, focusing on how these processes activate innate immunity and shape viral pathogenesis. In this mSphere of Influence article, she highlights how the work of Aguirre et al. (Nat Microbiol 2:17037, 2017, https://doi.org/10.1038/nmicrobiol.2017.37) changed her perception of how RNA viruses interact with pattern recognition receptors and how later the work of Wang et al. (Nature 616:152–158, 2023, https://doi.org/10.1038/s41586-023-05851-w) shaped her concept of cell death in viral infections. Framing these findings in different models of pathogen detection by the innate immune system, she reflects on how her understanding of the role of damage in RNA virus infection has evolved throughout her career.

## COMMENTARY

How does the body recognize viral infection? How do viruses engage the immune system during replication? These seemingly simple questions led me to study pattern recognition, a concept first proposed by Charles Janeway, Jr., in 1989 (1). Under this model, pathogen detection by a host organism is mediated by germline-encoded “pattern recognition receptors” (PRRs), which discriminate self from non-self by sensing evolutionarily distinct molecules termed “pathogen-associated molecular patterns” (PAMPs). Numerous PAMPs and PRRs were characterized in the years that followed, establishing pattern recognition as a fundamental mechanism of the innate immune system. A dogma arose with these discoveries: PRRs recognized viruses by hallmark proteins or nucleic acids present in the viral genome and replication intermediates (2, 3). Entering graduate school in 2013 obsessed with RNA viruses, I was initially drawn to study the viral RNA PRR retinoic acid-inducible gene 1 (RIG-I) (4). When my thesis research led me to focus instead on the cytosolic DNA PRR, cyclic GMP-AMP synthase (cGAS) (5), I began to feel disconnected from the RNA virus world.

As it turned out, studying a DNA-specific PRR was not too far from RNA viruses at all. A conundrum in the field had emerged. An adaptor protein in the cGAS pathway, stimulator of interferon (IFN) genes (STING), was shown to control RNA virus infection, and multiple RNA viruses were found to antagonize STING (6–10). Why would a DNA sensing pathway be relevant in RNA virus infection? Several theories were tested—STING was reported to facilitate RIG-I signaling (7, 11–13), to control basal levels of antiviral IFN signaling in cells (14), to inhibit translation during RNA virus infection (13), and to respond to membrane perturbations (15). These investigations uncovered fascinating aspects of STING biology that curtailed RNA viruses. However, Ana Fernandez-Sesma’s group provided another intriguing explanation in the context of dengue virus (DENV) infection. In their 2017 paper (16), they detailed how the DENV protein NS2B targeted cGAS for degradation, showing that DENV inhibited not only STING but also cGAS (6). This indicated that blockade of this DNA sensing pathway was absolutely critical for infection. They further showed that cGAS bound mitochondrial DNA (mtDNA) in the cytosol during infection to activate STING and downstream antiviral IFN signaling and corroborated previous reports of mitochondrial dysfunction in DENV-infected cells (17–19). Later confirmed by Eng Eong Ooi’s group (20), this was clear evidence that RNA virus replication damaged cells enough to generate DNA damage-associated molecular patterns (DAMPs) capable of activating a PRR-mediated antiviral response.

This finding indicated that RNA virus infection was subject to another model of innate immune sensing—the danger model. Not long after Janeway proposed that evolutionarily distinct PAMPs were sensed by PRRs to identify pathogens, Polly Matzinger proposed the danger model (21), which asserted that hosts must “detect and protect against danger.” Cellular damage poses such danger and is most readily considered critical in “sterile” diseases, such as autoimmune conditions and cancers (22). However, this work with DENV showed that cellular damage in viral infection also initiates innate immunity. Following this, multiple RNA viruses were shown to cause release of host mtDNA (23–25) and genomic DNA (gDNA) (26) to stimulate DNA-sensing PRRs. Indeed, these findings directly shaped my postdoctoral research by leading me to consider and then demonstrate that DNA DAMP release during severe acute respiratory syndrome coronavirus 2 infection triggered PRR-driven inflammation (27). Thus, RNA virus-induced damage has emerged as a mediator of innate immunity.

More recently, Jon Kagan posited that “infection infidelities” are key drivers of innate immunity during infection, in which PRRs sense the “mistakes” pathogens make (28). As examples of viral infection, Kagan suggested endosomal PRR activation by failed viral entry or cytosolic PRR activation by short, incomplete viral RNA elements, rather than activation of these PRRs by effective entry or viral genome replication. Though Kagan focused on the detection of evolutionarily distinct PAMPs, I cannot help but wonder how DAMPs from RNA virus infection fit into this model. Is damage a mistake that RNA viruses make? Many RNA viruses cause the lytic death of their host cell, a process linked to the release of DAMPs (29). Since a virus has limited potential mechanisms to curtail PRR activity outside of its replication cycle, is any form of host cell death-mediated DAMP release a mistake? This is further complicated by viruses that rely on cell lysis for virion release, which is true for many non-enveloped RNA viruses (30). A 2023 paper (31) from Tiffany Reese’s lab further highlights this conundrum by showing that a norovirus protein, NS3, caused cell death upon overexpression and that its N terminus had high homology to the key executioner of the necroptotic cell death pathway, mixed lineage kinase-like (MLKL). When phosphorylated, MLKL oligomerizes to form pores that insert into membranes to facilitate lytic cell death (32). Here, the Reese group demonstrated that NS3 formed oligomers and interacted with cardiolipin to disrupt mitochondrial membranes, leading to cell death. The authors further showed that this process led to virion release *in vitro* and *in vivo* and that viruses lacking the N terminus of NS3 or even just the mitochondrial localization motif within NS3 failed to cause fatalities in *Stat1^−/−^* mice, while wild-type viral infection did. This indicated that not only is cell death necessary for viral particle release but also is actively coordinated by a viral protein as a part of the replication cycle. Though the effect on the immune response remains unexplored, this process appears to be far from a mistake.

“It’s a feature, not a bug” is a half-joking refrain from programmers that reframes an unexpected function in an algorithm as an intentional design choice. While the concept of choice is problematic for RNA viruses, I find the role of damage opaque in the algorithm of viral infection. If infection causes cellular damage and that damage activates PRRs, then the immune system is activated, ideally leading to control of infection. This perspective makes it seem clear that damage must be a bug, a cost of viral replication. However, the paper from the Reese group and other recent publications point toward cell death and damage processes as key to at least some viral life cycles (33–36). This suggests that damage can be a feature of viral infection necessary for replication. Therefore, the question remains—is damage in RNA virus infection a feature or a bug? Though far from straightforward, the answer likely shapes viral disease. If host-derived damage is relevant in both viral replication and the immune response, then the host becomes an active participant in disease progression, regardless of its purpose. This may help explain why disease caused by RNA viruses varies so greatly in addition to genetic differences between hosts. As I set up my laboratory last year, I was guided by my fascination with this intrinsically chaotic aspect of the host-pathogen interface. Its interrogation will provide critical new insights into viral immunity, evolution, and pathogenesis.
